# Sleep disordered breathing and neurobehavioral deficits in children and adolescents: a systematic review and meta-analysis

**DOI:** 10.1186/s12887-023-04511-2

**Published:** 2024-01-20

**Authors:** Weiyu Zhang, Yubin Shen, Xiwen Ou, Hongwei Wang, Song Liu

**Affiliations:** Department of Respiratory Medicine and Sleep Lab, Xinhua Hospital, School of Medicine, Shanghai Jiao Tong University, Shanghai, 200092 China

**Keywords:** Sleep disordered breathing, Behavior disorders, Mood disorders, Children, Adolescents, Population attributable fraction, Meta-analysis

## Abstract

**Background:**

Sleep disordered breathing (SDB) is broadly recognized to be associated with neurobehavioral deficits, which have significant impacts on developing-aged children and adolescents. Therefore, our study aimed to quantify the proportion of neurobehavioral impairments attributed to SDB in general children and adolescents by population attributable fraction (PAF).

**Methods:**

The study was registered at PROSPERO (ID: CRD42023388143). We collected two types of literature on the prevalence of SDB and the risk of SDB-related neurobehavioral deficits from ten electronic databases and registers, respectively. The pooled effect sizes (P_e_, P_c_, RR) by random-effects meta-analysis were separately substituted into Levin’s formula and Miettinen’s formula to calculate PAFs.

**Results:**

Three prevalence literature and 2 risk literature, all with moderate/high quality, were included in the quantitative analysis individually. The prevalence of SDB was 11% (95%CI 2%-20%) in children and adolescents (P_e_), while the SDB prevalence was 25% (95%CI 7%-42%) in neurobehavioral patients (P_c_). SDB diagnosis at baseline was probably associated with about threefold subsequent incidence of neurobehavioral deficits (pooled RR 3.24, 95%CI 1.25–8.41), after multi-adjustment for key confounders. Up to 19.8% or 17.3% of neurobehavioral consequences may be attributed to SDB from Levin’s formula and Miettinen’s formula, respectively.

**Conclusions:**

A certain number of neurobehavioral consequences may be attributable to SDB. It is essential for clinicians to identify and treat SDB timely, as well as screen for SDB in patients with neurobehavioral impairments. More longitudinal studies of SDB and neurobehavioral deficits are needed in the future to further certify the association between them.

**Supplementary Information:**

The online version contains supplementary material available at 10.1186/s12887-023-04511-2.

## Background

Obstructive sleep disordered breathing (SDB) encompasses a spectrum ranging from primary snoring to upper airway resistance to obstructive sleep apnea [1]. At the worst end, obstructive sleep apnea (OSA) is a disorder characterized by complete or partial obstruction of the upper airway and is associated with blood gas changes and abnormal sleep patterns [2]. At the mild end, primary snoring (PS) is defined as habitual snoring in the absence of apnea, hypopnea, frequent arousals, or abnormal gas exchange [3].

Neurobehavioral deficits are series of potential brain-mediated dysfunctions [4] which may lead to cognitive, behavioral, and emotional abnormalities [5]. Children and adolescents are at a developmental age experiencing physical and neurobehavioral changes and synaptic remodeling processes, which mainly occur while sleeping [6]. Therefore, diseases during sleep may cause abnormal neurobehavioral development in this period [7–9]. Our studies focus on the relationship between SDB and neurobehavioral defects in the developmental stage. SDB not only has been associated with neurocognitive [10–16] and behavioral [12–14, 17–26] deficits, but also has been linked to mood disorders [27–30]. Sleep fragmentation and intermittent hypoxia are the two main pathophysiological pathways of SDB [31], with the former potentially showing the most impact on behavior, whereas the latter may have a more significant effect on cognition [32, 33]. The underlying mechanisms of SDB and mood disorders remain unclear. Depression is one of the most widespread mood disorders [29]. Previous studies have proposed that impaired daytime functioning (cognitive impairments, inattention, behavior problems, etc.) [3, 34–36] and damaged brain areas (bilateral hippocampus and caudate nucleus, white matter) [37, 38] caused by SDB, as well SDB-related serotonin transporter gene [39, 40] may increase the risk of depression, but further studies are needed to support these hypotheses.

Methodologically, the longitudinal association between SDB and neurobehavioral deficits has been measured using the odds ratio (OR) [23] and the hazard ratio (HR) [30], which have demonstrated the elevated risk of neurobehavioral impairments in individual children and adolescents with SDB. OR and HR are efficient measures of individual risk for neurobehavioral deficits in children and adolescents with SDB, but they cannot assess the expected new cases in a specific time window in the general population [41]. To infer the overall risk of the general population from the individual risk, population attributable fraction (PAF) is an appropriate epidemiological tool [42], which is defined as the proportion of risk reduction in an outcome over a specified time interval after elimination of exposure, while the distribution of other risk factors stays stable [43, 44].

To our knowledge, there is no published meta-analysis using PAF to quantify the proportion of neurobehavioral consequences attributable to SDB in children and adolescents. This study aimed to evaluate the risk of neurobehavioral impairments attributed to SDB in the general children and adolescents’ population. We extracted SDB prevalence in the general population and neurobehavioral cases, as well as the risk associated with SDB-related neurobehavioral deficits from the prevalence literature and the risk literature, respectively. Then combining these data by two different PAF formulas (Levin’s formula and Miettinen’s formula [45]) for analysis. The PAF estimates the percentage of neurobehavioral effects attributable to SDB in the general population of children and adolescents within a specific time window and is designed to pay more attention to patients with undiagnosed and/or untreated SDB to prevent the long-term consequences of neurobehavioral deficits in the future.

## Methods

### Study design and registration

This systematic review and meta-analysis were registered at PROSPERO (ID: CRD42023388143). The Preferred Reporting Items for Systematic Reviews and Meta-Analysis (PRISMA) statement [46] was used to standardize the process of literature search, data extraction, results summary, and presentation.

### Inclusion criteria

For the data on SDB prevalence, the following inclusion criteria need to be considered: 1) general population of school-aged children and adolescents; 2) participants aged between 5 and 18 years old; 3) full nighttime polysomnography or overnight, limited-channel home cardiorespiratory recordings [21] to recognize SDB; 4) were cross-sectional studies; 5) full-text articles with original statistics.

The followings are the inclusion criteria of articles concerning the risk of neurobehavioral morbidity linked to SDB: 1) subjects aged between 5 and 18 years old; 2) used polysomnography or cardiorespiratory recordings to diagnose SDB; 3) diagnosed the control group as a non-SDB population; 4) appropriate measure for neurobehavioral deficits; 5)were case–control studies or cohort studies; 6)only full-text articles were included.

### Information sources and search strategy

We searched the following English and Chinese databases: PubMed, Embase, Cochrane, Web of Science, China Biology Medicine disc (CBMdisc), Wanfang Database, VIP Database for Chinese Technical Periodicals, and China National Knowledge Infrastructure (CNKI). Moreover, if there were unpublished statistics from ongoing trials would be obtained from the Chinese Clinical Trial Registry and ClinicalTrials.gov. The electronic searches mentioned above were up to January 4, 2023. We used two kinds of strategies to retrieve the prevalence literature and the risk literature respectively, which combined the Mesh terms and keywords. The search strategy for the prevalence literature was "Sleep Apnea Syndromes" AND "Prevalence", while for the risk literature was "Sleep Apnea Syndromes" AND ("Cognitive Dysfunction" OR "Mental Disorder" OR "Mood Disorders" OR " Neuropsychological ") AND "Risk" (the complete search strategy for Pubmed is shown in Additional file 1).

### Study selection

Duplicate records and ineligible article types (reviews, conference abstracts, meta-analyses, etc.) were removed automatically by Endnote 20 before the screening. Titles and abstracts of the remaining articles were screened by two independent researchers (Weiyu Zhang and Yubin Shen) to select the eligible literature for full-text assessment (95% agreement). In the full-text screening, two authors independently reviewed and included the qualified studies, irrespective of language (96% agreement). All the disagreements were resolved through adequate discussion with the third researcher (Song Liu).

### Data extraction

The information concerning two types of literature (the prevalence of SDB and the risk of neurobehavioral morbidity associated with SDB) was extracted separately in Excel by two independent investigators (Weiyu Zhang and Yubin Shen). The extracted data were as follows:

1. The prevalence of SDB
  1. Characteristics of the studies (first author’s name, publication year, and country).
  2. Characteristics of the participants (age and proportion of males).
  3. Sample size and the number of SDB patients in each study.
  4. Diagnostic methods and criteria for SDB.
2. The risk of neurobehavioral morbidity associated with SDB
  1. Characteristics of the studies (first author’s name, publication year, country, and study design type).
  2. Characteristics of the subjects (age and population origin).
  3. Primary outcomes and follow-up time in each study.
  4. Covariates adjusted in each study.
  5. Sample size and the number of subjects with primary outcomes in each group (SDB group and control group).

When the necessary information was not available, we contacted the authors by e-mail and if there was no response within three months, those articles will not be included in the meta-analysis.

### Data analysis and epidemiological statistical method

Review Manager 5.4 and Stata 14.0 were used for all data analyses below. For these two types of studies (prevalence and risk), the proportion was chosen as an effect size to represent prevalence, while RR was an appropriate measure to quantify risk, as all the risk studies we included were cohort study designs. Considering various definitions [47] and diagnostic criteria [48, 49] of SDB, as well as different assessments of outcome indicators that would affect prevalence and risk, the random-effects model was used for both types of studies. The generic inverse variance method was utilized for the estimation of all effect sizes. Furthermore, maximally covariate-adjusted estimates were conducted to minimize the confounding effects on the relationship between SDB and neurobehavioral impairments in the risk literature. After the two types of studies had weighted combinations separately, overall effects sizes and their 95% confidence intervals (95% CI) were presented.

Heterogeneity was tested by the I^2^ statistic and classified as low (25%), moderate (50%), and high (75%) [50]. Subgroup analysis and meta-regression are two commonly used to explore the sources of heterogeneity [51]. Because of insufficient studies included, only the prevalence literature was able to carry out subgroup analysis and meta-regression. To avoid data-driven analysis and the possibility of only reporting significant results selectively, we selected covariates in advance [52]. The covariates we initially considered for further analysis were race [53–56], BMI (body mass index) [6, 48, 56–63], the proportion of males [48, 57, 60, 62, 64], and diagnostic criteria [48, 49]. However, owing to the limited number of literature and missing essential data, only the meta-regression based on male proportion was ultimately performed.

Sensitivity analysis is a critical method to explore the impact of different studies on the outcomes and examine the robustness of the results. The principle of Stata’s sensitivity analysis is to exclude each study in turn to test its impact on the summary results. If the results maintain consistency across the re-analysis, holding robustness can be considered [65]. Publication bias (PB) is a critical concern in meta-analysis. However, there were inadequate studies included to evaluate PB using various methods including funnel plots, Egger’s regression test, and trim-and-fill as originally planned [66].

There are different formulas to calculate PAF, and to provide more reasonable results, both Levin’s formula and Miettinen’s formula were selected. As for Levin’s formula, it is the only one listed in some epidemiology textbooks [67, 68], but bias may occur with this method in the case of confounding factors [43] in observational studies. Levin’s formula is expressed as follows: $\mathrm{PAF }= \frac{\mathrm{Pe }({\text{RR}}-1)}{\mathrm{Pe }({\text{RR}}-1)+1}$, the P_e_ (prevalence of exposure in the population) and RR were replaced in the formula obtained from the prevalence literature and risk literature, respectively. Compared to Levin’s formula, Miettinen’s formula is more suitable for general use [45]. Miettinen’s method is calculated as follows:$\mathrm{PAF }=\frac{\mathrm{ Pc }({\text{RR}}-1)}{{\text{RR}}}$, where P_c_ represents the prevalence of SDB in the neurobehavioral patients in this article. That is to say, the percentage of people with SDB to the total numbers suffering from neurobehavioral impairments in both groups (SDB group and control group) is the prevalence of SDB in the neurobehavioral patients. Both P_c_ and RR were obtained from the risk literature.

### Assessment of study quality

Owing to all prevalence studies included were cross-sectional designs, while risk studies were cohort study designs, Agency for Healthcare Research and Quality (AHRQ) [69] and Newcastle–Ottawa Scale (NOS) [70] were used to assess the quality of studies respectively [71]. The AHRQ consists of eleven specific items (see Additional file 2 for more details), each of which counts as 1 point, with a maximum score of 11 points for each study. Article quality assessed by AHRQ is classified into three categories: low (0–4), moderate (5–8), and high (9–11) quality [72]. As for the NOS, it includes three domains of selection, comparability, and outcome, which are divided into eight specific items (see Additional file 3 for more details). In addition to the comparability item, which has 2 points, all the rest items are 1 point. The maximum score for each study is 9 points and study scores of 0–4, 5–6, and ≥ 7 were considered low, medium, and high quality, respectively [73]. Review Manager 5.4 was used for the quality assessment above.

## Results

### Study selection

**Fig. 1:**
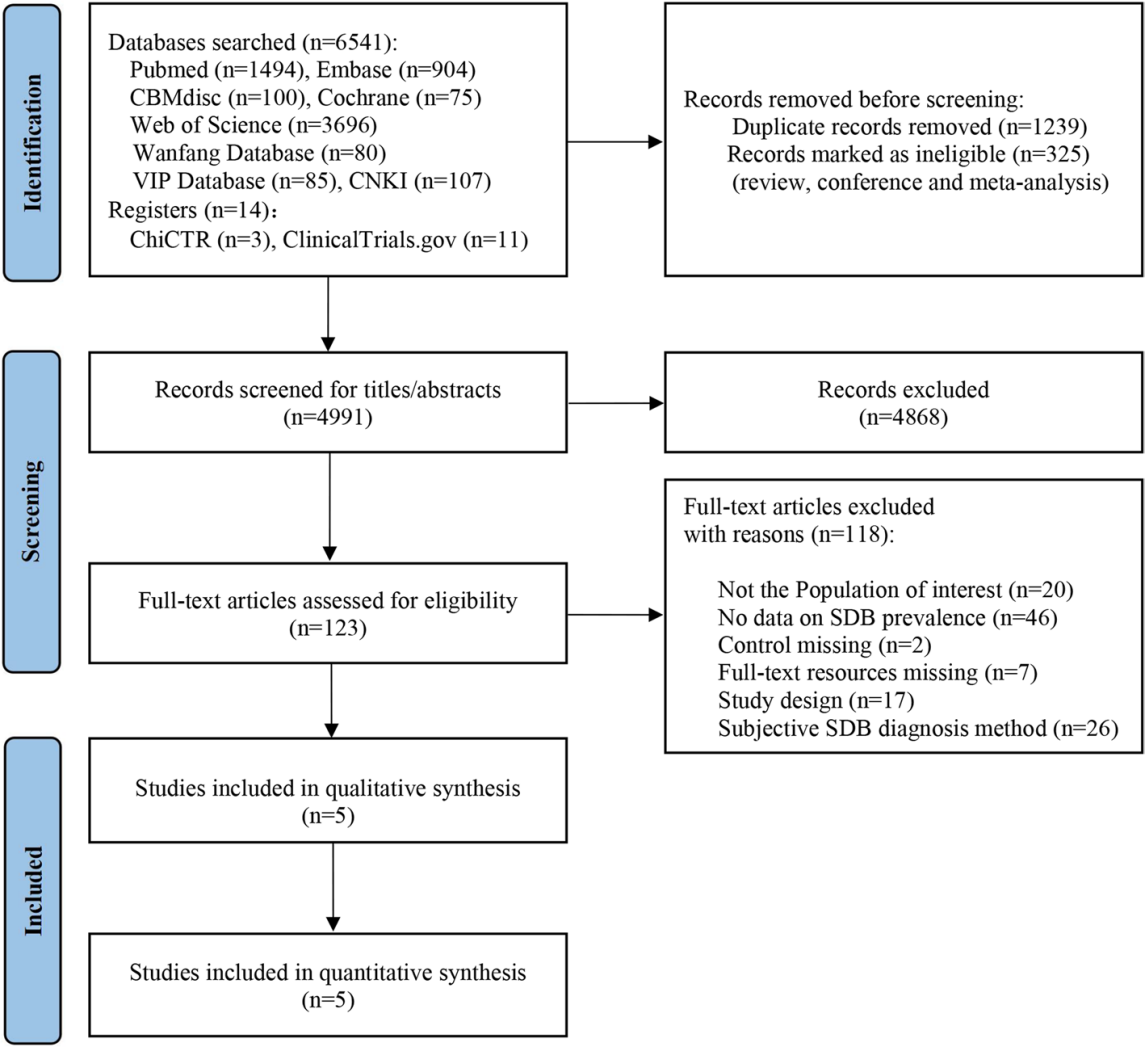
Flow diagram of the study selection process. Abbreviations: CBMdics: China Biology Medicine disc, VIP Database: VIP Database for Chinese Technical Periodicals, CNKI: China National Knowledge Infrastructure, ChiCTR: the Chinese Clinical Trial Registry

A total of 6,555 records were retrieved. Before the screening, 1564 were removed automatically by Endnote 20 for reasons of duplicate records and ineligible article types. The remaining 4991 articles were filtered by titles and abstracts, of which 123 articles were accepted into the full-text view. By scanning the full text, 118 articles were excluded, with the most common reason for exclusion being a lack of reported prevalence of SDB. Finally, three articles for SDB prevalence [21, 74, 75] and two for risk of neurobehavioral deficits [23, 30] linked to SDB were included in the meta-analysis. A detailed flow diagram of the study selection process is presented in Fig. 1.

### Study characteristics

As for the SDB prevalence studies, the characteristics are presented in Table 1. Among the three studies, two studies [21, 75] were conducted in the United States and the remaining one [74] was undertaken in Germany. Two studies [74, 75] diagnosed SDB by questionnaires and polysomnography (PSG), and one [21] study used cardiorespiratory recording, which had good agreement with PSG for the diagnosis of SDB [54]. Various definitions of SDB may contribute to different prevalence [47]. As the SDB in all risk literature we included did not include PS, the SDB in our study did not include PS to make the pooled data more uniform and representative. Excepting PS, C. L. Rosen et al. [21] defined OSA as AHI ≥ 5 or AI ≥ 1, E. O. Bixler et al. [75] categorized SDB as 1 ≤ AHI < 5 and AHI ≥ 5, and P. E. Brockmann et al. [74] classified SDB into OSA (AHI ≥ 1), UARS (AHI < 1 and RDI ≥ 1).

**Table 1:** Characteristics of the SDB prevalence studies

Author	Country	Sample size	Age (years)	Proportion of males
C. L. Rosen et al. [21] (2004)	United States	829	8.7–10.3	49.9%
E. O. Bixler et al. [75] (2009)	United States	700	5–12	47.8%
P. E. Brockmann et al. [74] (2012)	Germany	1144	8.9–10.3	47.2%
Author	SDB diagnosis method	SDB diagnosis Criteria	AHRQ scale (max 11)	
C. L. Rosen et al. [21] (2004)	Questionnaire Cardiorespiratory recording	OSA: AHI ≥ 5 or AI ≥ 1	8	
E. O. Bixler et al. [75] (2009)	Questionnaire PSG	1 ≤ AHI < 5 AHI ≥ 5	7	
P. E. Brockmann et al. [74] (2012)	Questionnaire PSG	OSA: AHI ≥ 1 UARS: AHI < 1 RDI ≥ 1	8	

*Abbreviations*: *OSA* obstructive sleep apnoea, *AHI* apnoea hypopnea index, *AI* apnea index, *PSG* polysomnography, *UARS* upper airway resistance syndrome, *RDI* respiratory disturbance index

Study characteristics of the risk of neurobehavioral impairments linked to SDB are illustrated in Table 2. One [30] is a retrospective cohort study from Chinese NHIRD, another [23] is a prospective cohort study from TuCASA of the United States. The main outcome measure in C. H. Chang et al. [30] was the occurrence of one or more depressive disorders, and the number of participants with depressive disorders in the SDB and control groups was 14/567 (2.47%) and 63/5670 (1.11%), respectively. While M. M. Perfect et al. [23] assigned the presence of behavioral impairment as the primary outcome measure. The Behavioral Symptoms Index (BSI) provides a broad combination of overall problem behaviors [76] in the Behavior Assessment Scale for Children Parent Report Form-2nd Edition (BASC-PRF), for which an abnormal value may indicate the presence of behavioral disorders. The number of those with behavioral disorders in the persistent SDB and never SDB groups was 6/17 (35.29%) and 10/135 (7.41%), respectively [23]. As for the covariates, one study [30] adjusted for age, sex, hypertension, diabetes, insomnia, ADHD, obesity, asthma, and Charlson comorbidity score, while another [23] only adjusted for sex, which was significant at the bivariate level in the BSI subscale. Follow-up periods ranged from 5 years to 5.87 years.

**Table 2:** Characteristics of the risk studies

Author	Country	Study design	Study population	Sample size	Age (years)
C. H. Chang et al. [30] (2017)	China	Retrospective cohort study	NHIRD	6237	9.7 ± 4.2
M. M. Perfect et al. [23] (2013)	United States	Prospective cohort study	TuCASA	152	13.25 ± 1.71
Author	Primary outcomes	The proportion of neurobehavioral deficits	Covariates	Observational period (years)	NOS scale (max 9)
C. H. Chang et al. [30] (2017)	One or more depressive disorders	SDB group: 14/567 (2.47%) Control group: 63/5670 (1.11%)	Age, sex, hypertension, diabetes, insomnia, ADHD, obesity, asthma, Charlson comorbidity score	5.87	8
M. M. Perfect et al. [23] (2013)	Behavioral impairment	SDB group: 6/17 (35.29%) Control group: 10/135 (7.41%)	Sex	5	7

*Abbreviations*: *NHIRD* National Health Insurance Research Database, *SDB* Sleep disordered breathing, *ADHD* Attention deficit hyperactivity disorder, *TuCASA* Tucson Children’s Assessment of Sleep Apnea Study

### Meta-analysis

#### The SDB prevalence in the general population

**Fig. 2:**
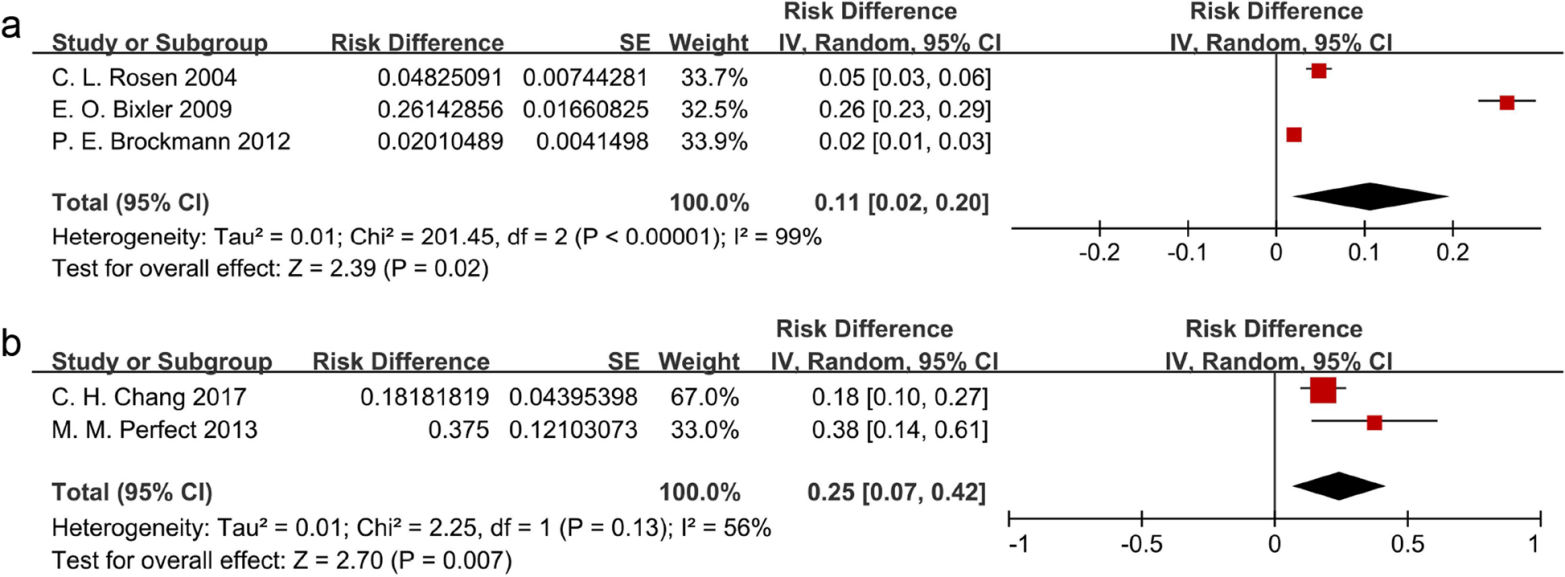
The SDB prevalence in the general population and neurobehavioral patients **a** The SDB prevalence in the general population; **b** The SDB prevalence in neurobehavioral patients; Black diamonds are the estimated pooled prevalence of SDB; red box sizes reflect the relative weight assigned to studies in the meta-analysis

The pooled prevalence is shown in Fig. 2. The weighted prevalence was 11% (95%CI 2%-20%). Across the three studies, high heterogeneity was detected (I^2^ = 99.0%). We further carried out a meta-regression based on the proportion of males, the result of which is presented in Additional file 4. The result shows *P* = 0.871 (95%CI -117.484–113.752), which suggests that the prevalence of SDB in this study was not significantly correlated with the proportion of males.

#### The SDB prevalence in neurobehavioral patients

The result of the weighted prevalence is presented in Fig. 2. The pooled prevalence was 25% (95%CI 7%-42%) in neurobehavioral patients. Moderate heterogeneity existed between the two studies (I^2^ = 56%).

#### The risk of neurobehavioral deficits associated with SDB

**Fig. 3:**
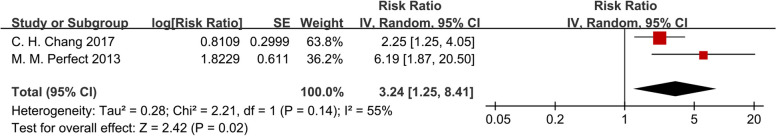
The risk of neurobehavioral deficits associated with SDB. Black diamonds are the estimated pooled risk ratio (RR); red box sizes reflect the relative weight assigned to studies in the meta-analysis

The results of the weighted synthesis are summarized in Fig. 3. Compared to non-SDB children and adolescents, patients with SDB had a three-fold higher risk of neurobehavioral impairments (overall RR 3.24, 95%CI 1.25–8.41), after multi-adjustment for the covariates mentioned above. There was moderate heterogeneity among the included studies (I^2^ = 55%).

#### Sensitivity analysis

The details of the sensitivity analysis are presented in Additional file 5. For the sensitivity analyses of the three effect sizes above (P_e_, P_c_, RR), the overall estimates were within the confidence interval after excluding each study individually. In other words, the results of these effect values (P_e_, P_c_, RR) had robustness.

### PAF of neurobehavioral deficits linked to SDB

Based on the three values (P_e_, P_c_, RR) obtained above, they could be respectively substituted into Levin’s formula and Miettinen’s formula to obtain two PAFs: PAF_1_ = 19.8% (Levin’s formula), PAF_2_ = 17.3% (Miettinen’s formula).

### Quality assessment

The quality ratings of the two types of literature (prevalence and risk) are revealed respectively in Tables 1 and 2 (see Additional file 6 for more details). As for the prevalence studies, the AHRQ scale was used for quality evaluation. The three studies scored from 7 to 8, all of which were moderate quality. NOS scale was applied to assess the quality of the risk literature and the two studies rated from 7 to 8, both of which had a low risk of bias.

## Discussion

This meta-analysis included three prevalence studies and two risk studies, the diagnosis of SDB at baseline was linked to a threefold incidence of neurobehavioral consequences, after multi-adjustment for critical confounders. Based on the results of PAF we have obtained by Levin’s formula and Miettinen’s formula, SDB in children and adolescents may contribute to a certain number of neurobehavioral impairments if the causal relationship can be proven definitely.

Due to multiple confounding factors (race, gender, socioeconomic factors, etc.) and mostly cross-sectional study designs, causality is challenging to ascertain, but it is now broadly recognized that SDB is related to neurobehavioral deficits [77]. As for the mechanisms linking SDB to cognitive impairments and behavioral disorders, the model of hypoxia/arousal interaction is the most broadly accepted mechanism for cognitive and behavioral deficits in children with SDB [77]. Confirmed indications from animal models suggest that hypoxic damage to the developing brain leads to long-term cognitive impairments [78, 79], but it is difficult to determine the extent of damage to the human brain developing from intermittent hypoxia [77]. There is a dose–response relationship between mild hypoxia, moderate hypoxia, and the level of mathematical impairment, which may be a manifestation of cognitive dysfunction, as shown by Urschitz et al. [80], while an inconsistent relationship exists across studies regarding the relationship between the severity of hypoxia and cognitive and behavioral function [81–83]. A study [84] has shown a corresponding improvement in electroencephalogram (EEG) slow wave activity (SWA) with improved oxygen saturation after the treatment of OSA. Since SWA is a marker of cortical development [85], it may provide some evidence for the relationship between repetitive hypoxia and brain development. As well, repeated arousals connected with respiratory events result in sleep disruption or fragmentation, which subsequently presents as excessive daytime sleepiness [77]. Unlike adults, children with sleepiness are at greater risk of showing behavioral deficits such as hyperactivity, mood deregulation, and oppositional behavior [86]. Depression is one of the most common mood disorders [29] and a type of emotional disorder in our included risk literature. Concerning the mechanism of the association between SDB and depression, there are some hypotheses accounting for the physiological changes caused by SDB that may contribute to depression, which need further studies to confirm [29]. Firstly, recurrent episodes of hypoxia caused by SDB damage the neurological system critically, including the bilateral hippocampus and caudate nucleus, and the white matter, leading to the development or progression of depression [37, 38]. Secondly, SDB may have daytime consequences, including cognitive deficits, inattention, behavior problems, and potentially lead to an increased likelihood of depressive symptoms [3, 34–36]. Lastly, OSA is associated with the serotonin transporter gene, which is engaged in susceptibility to depression [39, 40].

As there was significant heterogeneity (I^2^ = 99.0%) among the prevalence literature in our study, we further conducted pre-set meta-regression. We originally intended to perform the subgroup analysis based on diagnostic criteria and meta-regression depending on race (proportion of blacks), BMI, and male Proportion, but we included only three studies and all of them used different diagnostic criteria, as well as P. E. Brockmann et al. [74] who did not record race information, and the factor described by E. O. Bixler et al. [75] was BMI Percentile, which differed from others resulting in the inability to compare directly. Finally, meta-regression could only be conducted based on the proportion of males. The results showed *P* = 0.871 (95%CI -117.484–113.7517), indicating that the proportion of males was not related to the different prevalence across the studies. Overall, we have not found a potential source of heterogeneity in the prevalence literature so far. Nevertheless, all three effect sizes (P_e_, P_c_, RR) have robustness which may be considered reliable.

The PAF method allows us to assess not only the number of neurobehavioral impairments attributable to SDB but also the amount of avoidable neurobehavioral consequences theoretically. Due to a certain number of neurobehavioral deficits linked to SDB and its severe consequences, physicians should consider whether a newly diagnosed child with neurobehavioral impairments has SDB to provide timely treatment and improve the quality of life.

To the best of our knowledge, it is not only the first study quantifying new cases of neurobehavioral deficits attributable to SDB within a specific time window by using PAF, but also the first research to use Miettinen’s formula in the field of SDB. However, some limitations need to be acknowledged. First, we included a relatively small number of studies. As for the prevalence literature, PSG is the gold standard for the diagnosis of SDB, but it requires prolonged time, qualified operators, advanced devices, and high costs [63], which is an important reason why the literature on SDB diagnosis by objective methods is rare. When it comes to the risk literature, there are very few longitudinal studies regarding SDB and neurobehavioral deficits in children and adolescents, only the risk studies for behavioral and emotional dysfunction were included in this study as there was no suitable longitudinal study retrieved on the risk of cognitive disorders, resulting in an incomplete RR (obtained from the risk literature) in PAF. Second, we did not include the abstract-only and grey literature in our study, considering their reliability may not be well assessed. However, the exclusion may bias the results. Third, the pooled SDB prevalence may be underestimated to some extent. Because in the study of P. E. Brockmann et al. [74], only children with symptoms of habitual snoring were tested for PSG, which may have overlooked the presence of SDB in asymptomatic children. Fourth, to harmonize the included studies better, we had to exclude PS, but we do not deny the significant effects of PS. A review [77] suggests that the cognitive impairments in children with PS are largely similar to those with more severe OSA and even suffer from worse behavioral deficits. Fifth, the articles concerning the prevalence of OSA only were not be included. The purpose of our study was to obtain a comprehensive relationship between SDB and neurobehavioral disorders rather than solely focusing on OSA. Considering different types of SDB may have distinct pathophysiological mechanisms [87], we included a wider range of SDB to avoid the results being affected by OSA-specific characteristics or associated research biases, but we always value the importance of OSA. Sixth, neither source of heterogeneity has been found for the two types of literature (prevalence and risk) so far, and one of the reasons may be the few included studies, which prevented comprehensive subgroup analysis and meta-regression. Notably, the sensitivity analyses of the three effect sizes (P_e_, P_c_, RR) are robust, indicating that the results may be relatively reliable. Seventh, the PAF is an ideal but impractical measure but still represents a certain epidemiological value [88]. We utilized Levin's formula and Miettinen’s formula respectively to calculate the PAF. Of note, it is infeasible to estimate PAF unbiasedly by Levin’s formula in practice, as confounding factors are unavoidable in observational studies [89]. In the presence of confounding factors, Miettinen’s formula can also yield a valid PAF estimate if the adjusted RR is employed [90], but should be used with caution in meta-analysis [45]. In a word, both formulas have limitations in the application of meta-analysis and may lead to some bias. Even though the causality was confirmed, the PAF may overestimate the effect of SDB on neurobehavioral deficits, but the quantified PAF values still have a certain informative value. To some extent, it may indicate that a non-negligible amount of neurobehavioral impairments in children and adolescents can be attributed to SDB. Lastly, we could not assess publication bias with only two risk literature, but the relatively large effect size indicates that the inclusion of any missing studies is hard to invalidate the pooled association [88].

## Conclusions

The prevalence of SDB (exclude PS) is approximately 11% in children and adolescents, and the SDB patients at baseline may be associated with about threefold subsequent incidence of neurobehavioral deficits. This is not confirmable for a causal relationship between them as other confounding factors may remain. Of note, there is a certain number of neurobehavioral consequences that may be attributable to SDB, the PAFs derived from Levin’s formula and Miettinen’s formula are about 19.8% and 17.3%, respectively. This emphasizes the importance of early identification and treatment of SDB as well as screening for SDB in patients with neurobehavioral impairments for clinicians. More longitudinal studies of SDB and neurobehavioral deficits are needed in future work to further identify the association between them.

### Supplementary Information

**Additional file 1.**

** Additional file 2.**

** Additional file 3.**

** Additional file 4.**

** Additional file 5.**

** Additional file 6.**

## Data Availability

The datasets used and/or analysed during the current study are available from the corresponding author on reasonable request.

## Abbreviations

SDB: Sleep disordered breathing
PAF: Population attributable fraction
OSA: Obstructive sleep apnea
PS: Primary snoring
OR: Odds ratio
HR: Hazard ratio
PRISMA: Preferred Reporting Items for Systematic Reviews and Meta-Analysis
RR: Relative risk
CBMdisc: China Biology Medicine disc
CNKI: China National Knowledge Infrastructure
BMI: Body mass index
PB: Publication bias
AHRQ: Agency for Healthcare Research and Quality
NOS: Newcastle-Ottawa Scale
PSG: Polysomnography
BSI: Behavioral Symptoms Index
BASC-PRF: Behavior Assessment Scale for Children Parent Report Form-2nd Edition
EEG: Electroencephalogram
SWA: Slow wave activity
