# Supplementary material for: Sleep disordered breathing and neurobehavioral deficits in children and adolescents: a systematic review and meta-analysis
Source: BMC Pediatr. 2024 Jan 20;24:70. doi: 10.1186/s12887-023-04511-2 (PMC10799548; doi:10.1186/s12887-023-04511-2)
Supplement: Supplementary file 1 — Additional file 1. [file 12887_2023_4511_MOESM1_ESM.pdf]

Pubmed search strategy

1. Sleep Apnea Syndromes[MeSH Terms]
2. sleep apnea syndrome*[Title/Abstract]
3. sleep hypopnea*[Title/Abstract]
4. sleep apnea*[Title/Abstract]
5. mixed central and obstructive sleep apnea[Title/Abstract])
6. mixed sleep apnea*[Title/Abstract]
7. hypersomnia with periodic respiration[Title/Abstract]
8. sleep-disordered breathing[Title/Abstract]
9. sleep disordered breathing[Title/Abstract])
10. 1 or 2 or 3 or 4 or 5 or 6 or 7 or 8 or 9
11. Cognitive Dysfunction[MeSH Terms]
12. cognitive dysfunction*[Title/Abstract]
13. cognitive impairment*[Title/Abstract]
14. mild cognitive impairment*[Title/Abstract]
15. mild neurocognitive disorder*[Title/Abstract]
16. cognitive decline*[Title/Abstract]
17. mental deterioration*[Title/Abstract]
18. 11 or 12 or 13 or 14 or 15 or 16 or 17
19. Mental Disorders[MeSH Terms]
20. mental disorder*[Title/Abstract]
21. psychiatric illness*[Title/Abstract]
22. psychiatric disease*[Title/Abstract]
23. mental illness*[Title/Abstract]
24. psychiatric disorder*[Title/Abstract]
25. behavior disorders[Title/Abstract]
26. psychiatric diagnosis[Title/Abstract]
27. severe mental disorder*[Title/Abstract]
28. 19 or 20 or 21 or 22 or 23 or 24 or 25 or 26 or 27
29. Mood Disorders[MeSH Terms]
30. mood disorder*[Title/Abstract]

31. affective disorder*[Title/Abstract]

32. 29 or 30 or 31

33. Neuropsychological[Title/Abstract]

34. prevalence[MeSH Terms]

35. prevalence*[All Fields]

36. period prevalence*[All Fields]

37. point prevalence*[All Fields]

38. 34 or 35 or 36 or 37

39. Risk[MeSH Terms]

40. risk*[All Fields]

41. relative risk*[All Fields]

42. 39 or 40 or 41

43. 18 or 28 or 32 or 33

44. 10 and 38

45. 10 and 43 and 42

46. 44 or 45

47. 46 and (adolescent[Filter] OR child[Filter])
