# Supplementary material for: Sleep disordered breathing and neurobehavioral deficits in children and adolescents: a systematic review and meta-analysis
Source: BMC Pediatr. 2024 Jan 20;24:70. doi: 10.1186/s12887-023-04511-2 (PMC10799548; doi:10.1186/s12887-023-04511-2)
Supplement: Supplementary file 2 — Additional file 2. [file 12887_2023_4511_MOESM2_ESM.pdf]

S table- 1 Agency for Healthcare Research and Quality (AHRQ)

No.	Item
1	Define the source of information (survey, record review)
2	List inclusion and exclusion criteria for exposed and unexposed subjects (cases and controls) or refer to previous publications
3	Indicate time period used for identifying patients
4	Indicate whether or not subjects were consecutive if not population-based
5	Indicate if evaluators of subjective components of study were masked to other aspects of the status of the participants
6	Describe any assessments undertaken for quality assurance purposes (e.g., test/retest of primary outcome measurements)
7	Explain any patient exclusions from analysis
8	Describe how confounding was assessed and/or controlled
9	If applicable, explain how missing data were handled in the analysis
10	Summarize patient response rates and completeness of data collection
11	Clarify what follow-up, if any, was expected and the percentage of patients for which incomplete data or follow-up was obtained

1. Rostom A, Dubé C, Cranney A, Saloojee N, Sy R, Garritty C, et al. Appendix D. Quality Assessment Forms. Agency for Healthcare Research and Quality (US); 2004.
