# Supplementary material for: Sleep disordered breathing and neurobehavioral deficits in children and adolescents: a systematic review and meta-analysis
Source: BMC Pediatr. 2024 Jan 20;24:70. doi: 10.1186/s12887-023-04511-2 (PMC10799548; doi:10.1186/s12887-023-04511-2)
Supplement: Supplementary file 3 — Additional file 3. [file 12887_2023_4511_MOESM3_ESM.pdf]

NEWCASTLE - OTTAWA QUALITY ASSESSMENT SCALE
COHORT STUDIES

Selection

1) Representativeness of the exposed cohort

a) truly representative of the average ____(describe) in the community ✶

b) somewhat representative of the average ____in the community ✶

 c) selected group of users e.g. nurses, volunteers

 d) no description of the derivation of the cohort

2) Selection of the non exposed cohort

 a) drawn from the same community as the exposed cohort ✶

 b) drawn from a different source

 c) no description of the derivation of the non exposed cohort

3) Ascertainment of exposure

 a) secure record (e.g. surgical records) ✶

 b) structured interview ✶

 c) written self report

 d) no description

4) Demonstration that outcome of interest was not present at start of study

 a) yes ✶

 b) no

Comparability

1) Comparability of cohorts on the basis of the design or analysis

 a) study controls for ____(select the most important factor) ✶

 b) study controls for any additional factor ✶(This criteria could be modified to indicate specific control for a second important factor.)

Outcome

1) Assessment of outcome

 a) independent blind assessment ✶

 b) record linkage ✶

 c) self report

 d) no description

2) Was follow-up long enough for outcomes to occur

 a) yes (select an adequate follow up period for outcome of interest) ✶

 b) no

3) Adequacy of follow up of cohorts

 a) complete follow up - all subjects accounted for ✶

 b) subjects lost to follow up unlikely to introduce bias - small number lost- > __% (select an adequate %) follow up, or description provided of those lost) ✶

 c) follow up rate <___% (select an adequate %) and no description of those lost

 d) no statement

1. Wells G, Shea B, O'Connell D, Robertson J, Peterson J, Losos M, et al. The Newcastle-Ottawa Scale (NOS) for Assessing the Quality of Nonrandomized Studies in Meta- Analysis. Oxf UK. 2000.

2. Rostom A, Dubé C, Cranney A, Saloojee N, Sy R, Garritty C, et al. Appendix D. Quality Assessment Forms. Agency for Healthcare Research and Quality (US); 2004.
