# Supplementary material for: Sleep disordered breathing and neurobehavioral deficits in children and adolescents: a systematic review and meta-analysis
Source: BMC Pediatr. 2024 Jan 20;24:70. doi: 10.1186/s12887-023-04511-2 (PMC10799548; doi:10.1186/s12887-023-04511-2)
Supplement: Supplementary file 4 — Additional file 4. [file 12887_2023_4511_MOESM4_ESM.pdf]

Meta-regression based on the proportion of males

. metareg rate male, wsse (ser) bsest (reml) graph knapphartung

Meta-regression	Number of obs = 3
REML estimate of between-study variance	tau2 = .03323
% residual variation due to heterogeneity	I-squared_res = 99.48%
Proportion of between-study variance explained	Adj R-squared = -93.81%
With Knapp-Hartung modification	

S table- 1 Meta-regression based on the proportion of males

<table>
<tr><th>rate</th><th>Coef.</th><th>Std. Err.</th><th>t</th><th>P>|t|</th><th colspan="2">[95% Conf. Interval]</th></tr>
<tr><td>male</td><td>-1.866185</td><td>9.099323</td><td>-0.21</td><td>0.871</td><td>-117.484</td><td>113.7517</td></tr>
<tr><td>_cons</td><td>1.010941</td><td>4.396299</td><td>0.23</td><td>0.856</td><td>-54.84933</td><td>56.87121</td></tr>
</table>

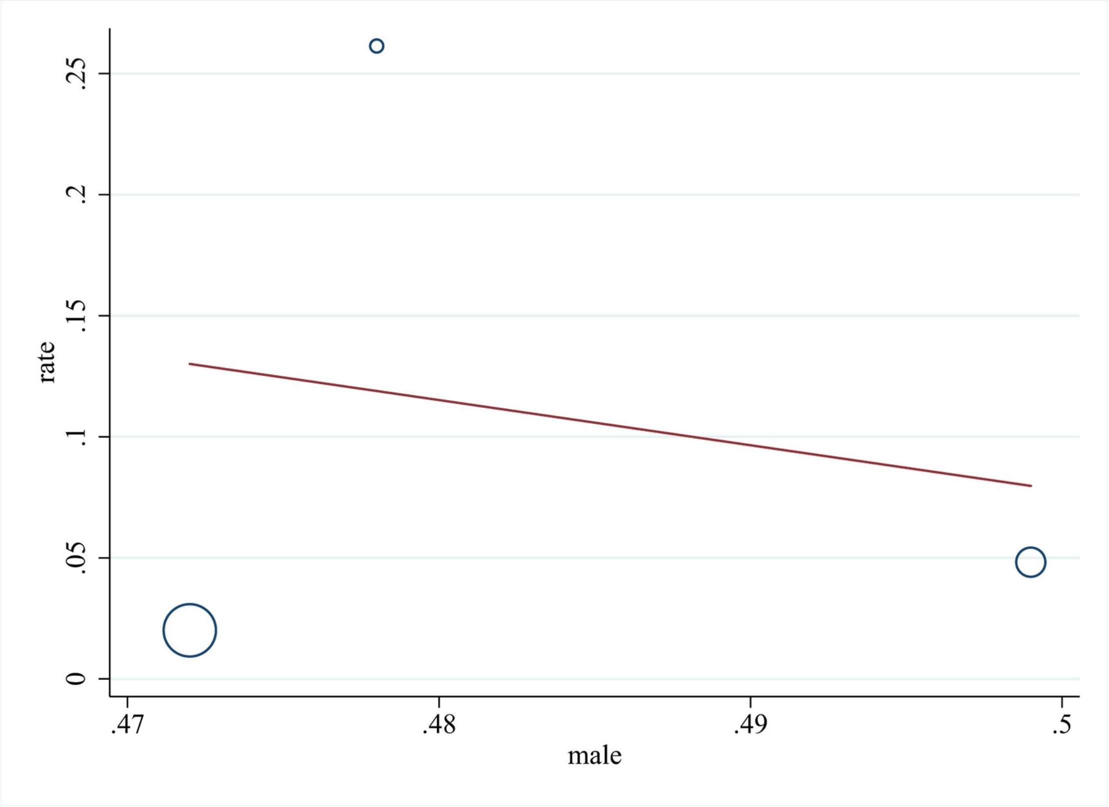

S figure- 1 Meta-regression based on the proportion of males
