# Supplementary material for: Sleep disordered breathing and neurobehavioral deficits in children and adolescents: a systematic review and meta-analysis
Source: BMC Pediatr. 2024 Jan 20;24:70. doi: 10.1186/s12887-023-04511-2 (PMC10799548; doi:10.1186/s12887-023-04511-2)
Supplement: Supplementary file 5 — Additional file 5. [file 12887_2023_4511_MOESM5_ESM.pdf]

The results of sensitivity analysis

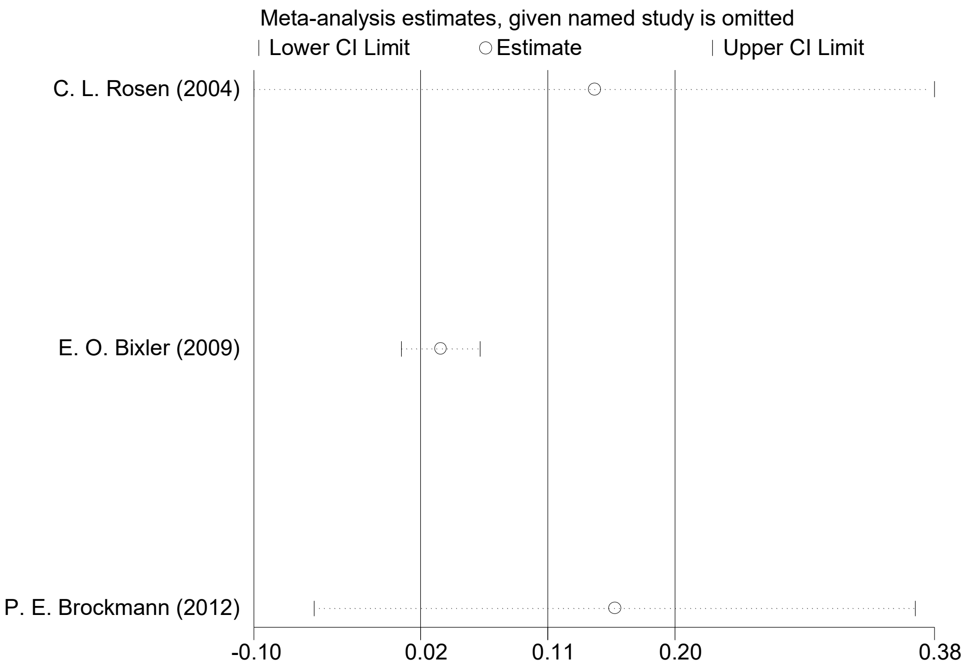

S figure-1 The sensitivity analysis of P_e

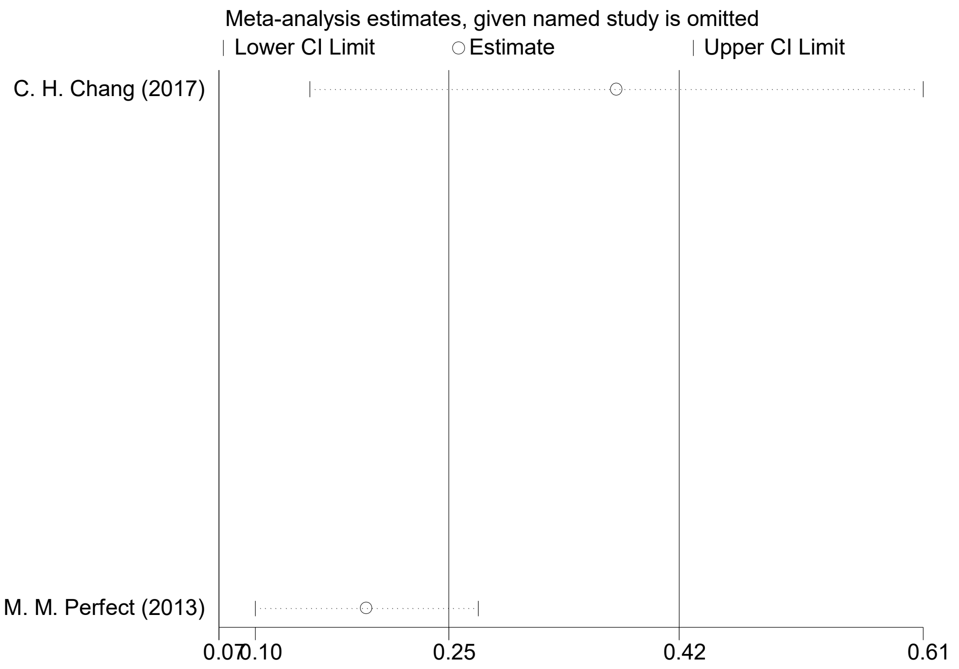

S figure-2 The sensitivity analysis of P_c

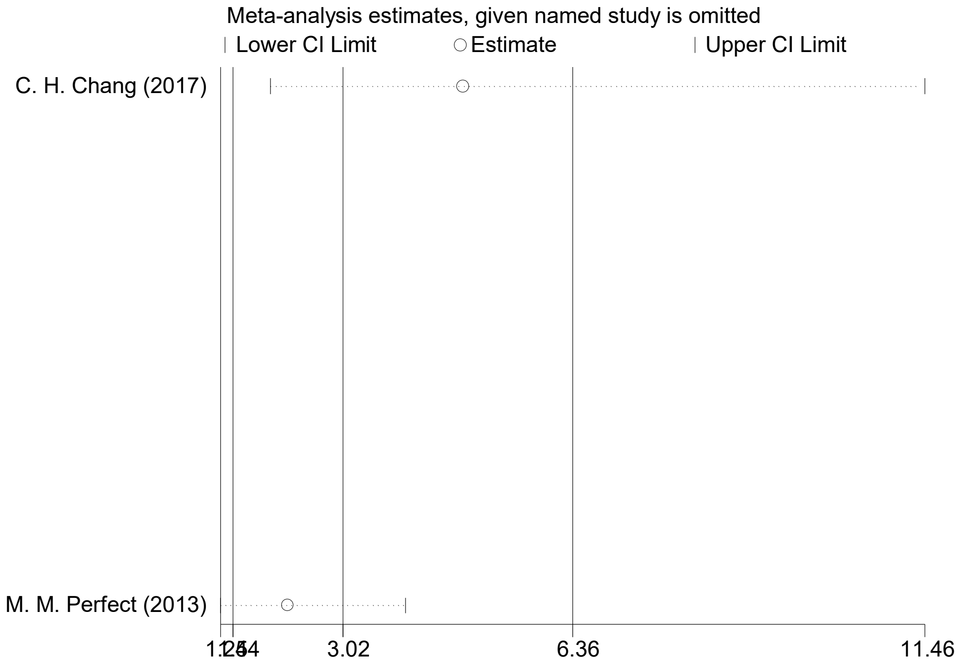

S figure-3 The sensitivity analysis of RR
