# Supplementary material for: Sleep disordered breathing and neurobehavioral deficits in children and adolescents: a systematic review and meta-analysis
Source: BMC Pediatr. 2024 Jan 20;24:70. doi: 10.1186/s12887-023-04511-2 (PMC10799548; doi:10.1186/s12887-023-04511-2)
Supplement: Supplementary file 6 — Additional file 6. [file 12887_2023_4511_MOESM6_ESM.pdf]

## The results of quality assessment

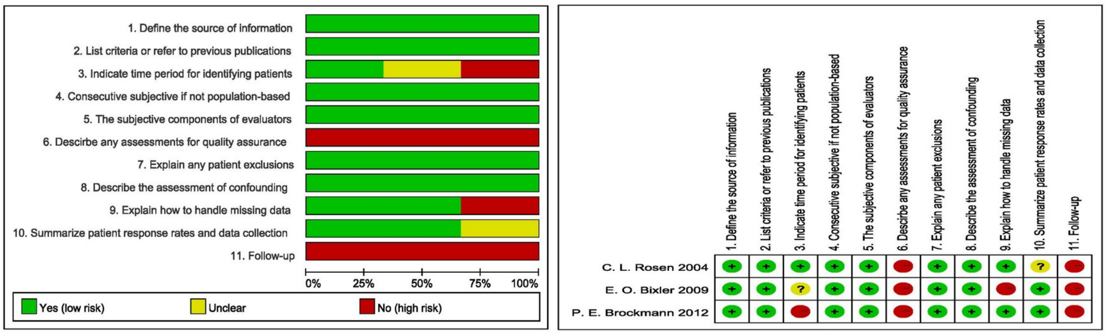


a b

S figure- 1 Quality assessment of the prevalence literature by AHRQ

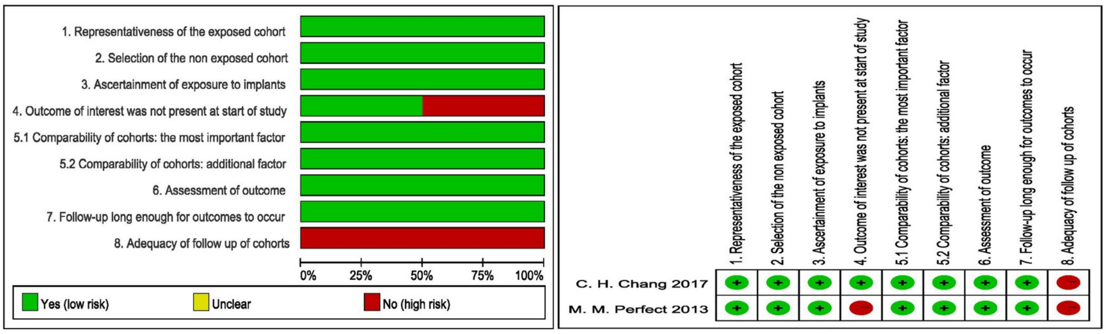


a b

S figure- 2 Quality assessment of the risk literature by NOS
